# Emerging Challenges in *Staphylococcus aureus* Bloodstream Infections: Insights from Coagulase Typing, Toxin Genes, and Antibiotic Resistance Patterns

**DOI:** 10.1155/2023/7041159

**Published:** 2023-12-23

**Authors:** Samira Zamani, Masoud Dadashi, Sara Bahonar, Mehrdad Haghighi, Sareh Kakavandi, Ali Hashemi, Mohammad Javad Nasiri, Bahareh Hajikhani, Mehdi Goudarzi

**Affiliations:** ^1^Department of Microbiology, School of Medicine, Shahid Beheshti University of Medical Sciences, Tehran, Iran; ^2^Department of Microbiology, School of Medicine, Alborz University of Medical Sciences, Karaj, Iran; ^3^Department of Infectious Diseases, Imam Hossein Teaching and Medical Hospital, Shahid Beheshti University of Medical Sciences, Tehran, Iran; ^4^Department of Bacteriology and Virology, School of Medicine, Shiraz University of Medical Sciences, Shiraz, Iran

## Abstract

**Background:**

The incidence of complications and mortality associated with *Staphylococcus aureus* (*S. aureus*) bloodstream infections has been increasing significantly, particularly in developing countries where control strategies against this virulent pathogen and its resistance to antibacterial agents are insufficient. The aim of this study was to investigate coagulase typing, the prevalence of toxin genes, and the antibiotic resistance profile of *S. aureus* isolated from bloodstream infections.

**Methods:**

Antibiotic susceptibility of the isolates was determined by the disk diffusion method. The prevalence of toxin genes was determined using the polymerase chain reaction (PCR) method. Genetic variability of isolates was determined using multiplex PCR based on coagulase gene polymorphism.

**Results:**

Out of 120 strains, 55 (46%) were methicillin-resistant *S. aureus* (MRSA) and 65 (54%) were methicillin-sensitive *S. aureus* (MSSA). All isolates were susceptible to linezolid and teicoplanin but showed varying levels of resistance to other antibiotics. The highest resistance was observed for ampicillin (92.5%), gentamicin (69.2%), and amikacin (68.3%). Multidrug resistance was observed in all isolates. PCR analysis revealed a higher prevalence of toxin genes in MRSA (*tst*: 38%, *pvl*: 29.1%, *eta*: 10%, and *etb*: 4.1%) than that in MSSA. According to the *coa* typing, the most prevalent types were *coa* III (29.2%), *coa* II (26.7%), and *coa* VI (10%).

**Conclusion:**

The presence of genetic variability and widespread multidrug resistance in our hospitals emphasizes the circulation of various *coa* types. Therefore, it is crucial to implement antimicrobial stewardship and infection control measures to prevent and control the spread of these strains.

## 1. Introduction

*Staphylococcus aureus* (*S. aureus*) is a Gram-positive, facultative anaerobe, nonmotile spherical-shaped bacterium associated with skin and soft tissue infections (SSTIs), osteomyelitis, pneumonia, bloodstream infections (BSIs), and life-threatening endocarditis [1–3]. Recent reports indicate an increased prevalence of *S. aureus* invasive infections, with bacteremia as a major clinical manifestation. *S. aureus* isolated from BSIs is now considered a high-priority pathogen with the potential to increase fatality rates globally [4–6]. The rising drug resistance in virulent strains, particularly methicillin-resistant *S. aureus* (MRSA), poses a serious challenge in the treatment and control of staphylococcal infections [7, 8]. The indiscriminate use of antibiotics in both the community and hospital settings has led to increased selective pressure on *S. aureus*, resulting in the proliferation of antibiotic resistance [7–9]. Evidences indicated that numerous *S. aureus* virulence factors including toxic shock syndrome toxin-1 (TST-1), panton-valentine leukocidin (PVL), staphylococcal enterotoxins (SEs), and exfoliative toxins (ETa and ETb) have been associated with the pathogenesis of this bacterium [10–12]. Several molecular typing methods have been employed to genotype *S. aureus* strains, including pulsed-field gel electrophoresis (PFGE), staphylococcal cassette chromosome *mec* (SCC*mec*) typing, *agr* typing, protein A gene (*spa*) typing, multilocus sequence typing (MLST), and coagulase gene (*coa*) typing [7, 13, 14]. *Coa* typing, a multiplex PCR-based method, is cost-effective, rapid, easily interpretable, and suitable for identifying genetic relationships among *S. aureus* isolates [15, 16]. Coagulase, an extracellular protein, plays a vital role in pathogenesis of *S. aureus* [16–19]. Generally, the activity of the coagulase enzyme in *S. aureus* pathogenesis is multifaceted. It aids in immune evasion, facilitates the establishment of infection, contributes to the formation of abscesses, and promotes biofilm formation. Understanding the role of coagulase is crucial for developing strategies to combat *S. aureus* infections.

Ten different *coa* types (ScI-X) have been identified based on variations in the signal sequence, N-terminal D1 and D2 regions, central region, 27-amino acid repeat regions, and C-terminal sequence [16, 20]. Recent studies have reported a correlation between specific genotypes of *S. aureus*, multidrug-resistant (MDR) patterns, and virulence profiles [2, 3, 6, 8, 9]. There are only a small number of studies available worldwide addressing the genotyping of *S. aureus* isolated from blood infections [4, 7]. The present study was attempted to determine the antibiotic resistance pattern, toxin profile, and molecular characteristics of *S. aureus* isolates obtained from patients with bacteremia.

## 2. Methods and Materials

### 2.1. Study Design, Sample Collection, and Ethical Considerations

In this cross-sectional study, from August 2021 to July 2022, a total of 120 *S. aureus* isolates obtained from BSIs were investigated. The study protocol received approval from the Ethics Committee of the Shahid Beheshti University of Medical Sciences in Tehran, Iran (IR.SBMU.MSP.REC.1401.714). All isolates were cultured on blood agar and mannitol salt agar and characterized by Gram staining and conventional biochemical tests, including catalase production, and coagulase and DNase tests (HiMedia, Mumbai, India). All phenotypically confirmed *S. aureus* isolates underwent polymerase chain reaction (PCR) assay for the *nuc* gene detection and final confirmation. All confirmed isolates were stored at −70°C in Tryptic Soy Broth (TSB, HiMedia, Mumbai, India) supplemented with 20% glycerol for further analysis. To screen for methicillin-resistant *S. aureus* (MRSA) strains, a disk diffusion method using cefoxitin (30 *μ*g) on Mueller–Hinton agar was performed. The *mecA* genes were detected by PCR as described elsewhere [21].

### 2.2. Antibiotic Susceptibility Determination

For antimicrobial susceptibility testing of the isolated *S. aureus*, the Kirby–Bauer disk diffusion method was applied using commercial antibiotics including amikacin (AMK, 30 *μ*g), tetracycline (TET, 30 *μ*g), erythromycin (ERY), kanamycin (KAN, 30 *μ*g), linezolid (LIN, 30 *μ*g), teicoplanin (TEI, 30 *μ*g), clindamycin (CLI, 2 *μ*g), gentamycin (GEN, 10 *μ*g), rifampicin (RIF, 5 *μ*g), ampicillin (AMP, 10 *μ*g), chloramphenicol (CHL, 30 *μ*g), cephazolin (CFZ, 30 *μ*g), ciprofloxacin (CIP, 5 *μ*g), and trimethoprim-sulfamethoxazole (SXT, 1.25/23.75 *μ*g) according to the Clinical Laboratory Standards Institute (CLIS, 2020) guidelines (Mast Co., Ltd., UK). MDR was specified as resistance to 3≥ classes of antibacterial agents as described earlier [8]. We employed ATCC 25923 (routine quality control) and ATCC 43300 (*mecA*-mediated resistance using cefoxitin) as quality control strains.

### 2.3. Evaluation of Toxin Gene Expression

The PCR technique with specific dinucleotide primers was employed to detect toxin-encoding genes, including panton-valentine leukocidin (*pvl*), exfoliative toxins (*eta*, *etb*), and toxic shock syndrome toxin (*tst*) (TIB Molbiol, Berlin, Germany). In each run test, positive controls for toxin genes were used.  Table 1 provides a description of the primers and amplification conditions utilized.

### 2.4. Coagulase Typing

The *coa* types (I–X) were analyzed using a multiplex PCR-based method with four sets (A–D) of primers and PCR conditions explained by Hirose et al. [16]. Set A primers were designed to identify *coa* types I, II, III, IVa, IVb, and Vb. Set B primers were utilized to distinguish *coa* types VII, VIII, and X. Set C primers were used for the identification of *coa* types IX and Vb. Finally, Set D primers differentiated between *coa* types IVa and IVb. As positive controls, standard *S. aureus* strains representing coa types I–X were used in each PCR assay.

### 2.5. Statistical Analysis

The data were analyzed with Statistical Package for Social Sciences (SPSS) software V22.0 for Windows. Qualitative data were expressed using numbers and percentages.

## 3. Results

### 3.1. Data and Antibiotic Susceptibility

In this study, a total of 120 *S. aureus* clinical isolates were collected from blood samples. The blood cultures were incubated for 18–24 hours, and the positive cultures were cultured on blood agar and mannitol salt agar. It is important to note that only one sample was obtained from each patient, ensuring that the isolates were nonduplicate. One sample was obtained from each patient. The exclusion criteria for this study involved the presence of bacterial species other than *S. aureus* in the blood cultures, as well as contaminated blood cultures. The mean age of patients was 46.6 years, ranging from 1 to 69 years old, with 47.5% (*n* = 57) being male and 52.5% (*n* = 63) being female. The occurrence of *S. aureus* bacteremia was more frequent in the age group 30–70, with the lowest occurrence observed in age groups under 10 and above 60. The distribution of *S. aureus* isolates in various hospital wards was as follows: intensive care unit (ICU) (38.3%; 46/120), infectious disease (20%; 24/120), cardiopulmonary (13.3%; 16/120), burn unit (22.5%; 27/120), nephrology (2.5%; 3/120), urology (2.5%; 3/120), and pediatric intensive care unit (0.9%; 1/120). MRSA strains were detected in 55 isolates (46%). The study showed that 65 out of 120 isolates (54%) were classified as MSSA. All tested strains were susceptible to teicoplanin and linezolid.

The antimicrobial susceptibility testing revealed the highest resistance rate for ampicillin (92.5%), followed by gentamicin (69.2%), amikacin (68.3%), erythromycin (61.7%), tetracycline (46.7%), kanamycin (39.2%), clindamycin (45.8%), chloramphenicol (20%), cephazolin (20%), ciprofloxacin (42.2%), rifampin (32.5%), and trimethoprim-sulfamethoxazole (13.3%) (Table 2). All of the isolates were found to be MDR based on the antimicrobial susceptibility testing. As given in Table 3, the most prevalent resistance profile among the MDR isolates was resistance to 3 antibiotics (24.2%), followed by resistance to 4 antibiotics (20.8%), 6 antibiotics (20%), and 10 antibiotics (11.4%) simultaneously. In our study, eleven resistance patterns were detected, wherein ERY, AMP, and AMK (8.3%) and AMP, CIP, CEF, GEN, ERY, and CLI (15%) were the top frequently identified profiles in MSSA and MRSA strains, respectively.

### 3.2. Distribution of Virulence Encoding Gene

In this study, the *tst* gene was confirmed in 38.3% of the isolates but the *pvl* gene was present in 29.2%, *eta* in 10%, and *etb* in 4.2% of tested isolates. The prevalence rates of toxin genes were higher in MRSA strains compared to MSSA strains. *S. aureus* isolates carrying *pvl* genes had resistance to 6 and 4 antibiotics simultaneously, while TST-positive isolates indicated resistance to 3, 4, and 6 antimicrobial agents.

### 3.3. Distribution of *coa* Types Based on Multiplex PCR

According to the multiplex PCR test for *coa* typing of 120 tested isolates, type III had highest prevalence, representing 29.2% (35/120), followed by types II (26.7%; 32/120), VI (10%; 12/120), VIII (9.1%; 11/120), I (7.5%; 9/120), X (5%; 6/120), VII (4.2%; 5/120), IVb (3.3%; 4/120), Iva (2.5%; 3/120), and Va (2.5%; 3/120) (Table 4). The highest prevalence rate of *pvl* and *tst* genes was observed in *S. aureus* strains with *coa* type II. Additionally, *eta* and *etb* genes were detected in *coa* types I and III.

## 4. Discussion

Microbial resistance of *S. aureus*, especially MRSA, is a serious problem for human health and patients worldwide. As MRSA causes bloodstream infections in hospitalized patients, the increased antibiotic resistance in this bacterium is a significant threat [22, 23]. Multidrug resistance has been recognized as a major issue in healthcare management due to the consequences of inadequate infection control measures [24]. Several studies have investigated the resistance rates of *S. aureus* isolates from blood samples [7–9], and the present study reports similar findings among patients with bacteremia at different wards of Shahid Beheshti University of Medical Sciences hospitals. The first aspect we assessed was the sensitivity of the isolates to linezolid, an advanced antimicrobial agent known for its activity against *S. aureus* isolates. In line with findings from previous research [24–26], the present study showed that all the isolates were sensitive to linezolid. This finding suggests that linezolid may be a potential treatment option for patients infected with MDR *S. aureus* strains [27].

There is considerable data on the prevalence rate of MRSA strains isolated from BSIs across the world. In line with our results, a high prevalence of MRSA strains was also reported in earlier research conducted in different geographical regions [22–25]. Our study revealed a high prevalence of resistance to many antibiotics commonly used for bloodstream infections (BSIs). Interestingly, no resistance to teicoplanin and linezolid was observed, consistent with findings reported by Alharbi [25]. Conversely, the highest resistance was detected against ampicillin and amikacin. Furthermore, all analyzed isolates were classified as MDR, indicating a particular risk to public health. These findings emphasize the urgent need for implementing appropriate antibiotic stewardship programs and the use of specific treatment protocols in patients infected with these strains to mitigate the spread of MDR *S. aureus*.

The study also reported a high percentage of MDR among MRSA strains compared to MSSA. Various studies from different regions have also reported similar findings [28, 29]. A similar survey conducted by Eslami et al. on 59 *S. aureus* isolated from wound showed that the prevalence of MRSA (59.3%) was greater than that of MSSA (40.7%) [13]. Soltani et al. recently performed an epidemiological analysis on 95 *S. aureus* and investigated the MDR in MRSA and MSSA strains isolated from different clinical samples. They reported a higher MDR rate in MRSA strains compared to MSSA (87.5% vs 77.4%) [3]. A study by Rahimi et al. focused on 419 clinical *S. aureus* strains isolated from urinary tract infection. They indicated that 25.8% and 74.2% of tested isolates were MRSA and MSSA, respectively. Also, all the strains were to be susceptible to linezolid, vancomycin, penicillin, and tigecycline [11]. Recent evidence has highlighted the pivotal role of toxins as a major virulence factor in the pathogenesis of *S. aureus*-induced infections [26]. According to our data, 38.3%, 29.2%, 10%, and 4.1% of *S. aureus* strains were found to carry *tst*, *pvl*, *eta*, and *etb* encoding genes, respectively. This result is similar to that of the Iranian study by Goudarzi et al., who indicated that the *tst* gene was the predominant type in *S. aureus* strains (58.6%), followed by *pvl* (19.5%), *eta* (7.8%), and *etb* (5.5%), respectively [8]. A study conducted by NI Ahmad et al. [28] in Malaysia reported that only 20% of MRSA strains were positive for the *pvl* gene, which was in contrast with our results. However, similar to our findings, the prevalence rate of the *pvl* gene in MRSA strains was higher than that in MSSA strains. Differences in the prevalence of the *pvl* gene among *S. aureus* strains may be influenced by factors such as origin of isolates, clinical samples, and geographical factors and, most importantly, the presence of diverse PVL-carrying phages within *S. aureus* strains.

Our results, in relation to *tst* gene, coincide with those produced by Soltani et al. in Iran who reported *tst* gene in 18.9% of *S. aureus* strains isolated from clinical samples [3]. Similarly, in a systematic review and meta-analysis study conducted in Iran, Shahini Shams-Abadi et al. reported a relatively high carriage rate of *tst* gene (21.3%) [29]. In a similar survey conducted in Korea by Kim et al., on 576 *S. aureus* strains isolated from BSIs, showed that 26.8% of the examined isolates were *tst* positive [30]. Possible explanations for this raised prevalence are the genetic diversity of bacteria, high expression levels of this gene in BSIs, and the transmission of these genes among different isolates.

In our study, the prevalence of the *eta* and *etb* genes was found to be relatively low, consistent with the findings reported by Tayebi et al. [31]. This indicates that the presence of these exfoliative toxin genes may not be significantly associated with bloodstream infections. Furthermore, the low prevalence of these exfoliative toxin genes may suggest that other more virulent factors are contributing to bloodstream infections in this population.

As presented in Table 4, 10 different *coa* types are detected in this research. In agreement with the published data by Hirose et al. [17], which indicated genetic variability of *S. aureus* in Japan, the current research indicated genetic diversity of *S. aureus* isolated from blood infection with the predominant genotype of *coa* III (29.2%). Likewise, a study by Afrough et al., conducted in Iran, examined 157 clinical isolates of *S. aureus* and revealed a high prevalence rate of MRSA (83.7%), as well as genetic diversity in *coa* genes. The study identified six different *coa* patterns, with the C1 pattern being the most common (21.7%) [18]. Another study conducted by Goudarzi et al. in Iran, on 89 MRSA strains from burn patients, indicated 10 *coa* types with the majority of type III (47.2%) [32]. A similar survey conducted by Mohajer et al. in Iran on 258 *S. aureus* isolates indicated five *coa* types [33]. In an Egyptian study conducted by Abdulghani and Khairy on 58 MRSA strains recovered from clinical isolates, 15 different *coa* types with the prominence of *coa* type X (16.6%) were identified [34]. In a Sudanese study conducted by Ibrahim et al., resistance to methicillin was found to be 56% and *coa* type III as the predominant type was reported (55.5%) [35].

## 5. Conclusion

This study provides valuable insights into the prevalence of antibiotic resistance, toxin gene expression, and coagulase types among *S. aureus* strains causing bacteremia. The genetic diversity of the *coa* gene, with the prominence of *coa* type III and high MDR, poses potential risks in our healthcare settings, emphasizing the need for proper and comprehensive approaches for systematic surveillance to reduce the rate of infections. Continuous monitoring of the antimicrobial susceptibility surveillance system and antibiotic stewardship programs (ASPs) are necessary for better patient management. The observed genetic variability highlights the significant health risk posed by *S. aureus* in patients with bacteremia.

## Figures and Tables

**Table 1:** The PCR conditions and oligonucleotide primers used in this study.

Gene	Primer	Sequence	*PCR condition (35 cycles)*				Reference
			Denaturation (seconds)	Annealing (seconds)	Extension (seconds)	Product size (bp)	
*fem*	F	CTTACTTACTGCTGTACCTG	94°C, 50	55°C, 60	72°C, 50	648	[1]
	R	ATCTCGCTTGTTGTGTGC					

*nuc*	F	GCGATTGATGGTGATACGGTT	95°C, 45	56°C, 50	72°C, 55	270	[2]
	R	AGCCAAGCCTTGACGAACTAAAGC					

*mecA*	F	AGAAGATGGTATGTGGAAGTTAG	94°C, 45	55°C, 50	72°C, 50	583	[1]
	R	ATGTATGTGCGATTGTATTGC					

*pvl*	F	TTCACTATTTGTAAAAGTGTCAGACCCACT	95°C, 60	57°C, 60	72°C, 60	180	[3]
	R	TACTAATGAATTTTTTTATCGTAAGCCCTT					

*tst*	F	TTATCGTAAGCCCTTTTGTTG	95°C, 60	56°C, 60	72°C, 60	398	[3]
	R	TAAAGGTAGTTCTATTGGAGTAGG					

*eta*	F	GCAGGTGTTGATTTAGCATT	94°C, 45	57°C, 45	72°C, 50	93	[2]
	R	AGATGTCCCTATTTTTGCTG					

*etb*	F	ACAAGCAAAAGAATACAGCG	94°C, 45	56°C, 45	72°C, 55	226	[2]
	R	GTTTTTGGCTGCTTCTCTTG					

F: forward; R: reverse. For all samples, initial denaturation of 95°C for 5 minutes and final extension of 72°C for 5 minutes were performed.

**Table 2:** Antibiotic resistance pattern of 120 *S. aureus* isolates collected from blood samples.

Antibiotics	MRSA, *n* (%)	MSSA, *n* (%)	Total resistance, *n* (%)
Ampicillin	70 (63.1)	41 (36.9)	111 (92.5)
Amikacin	45 (54.9)	37 (45.1)	82 (68.3)
Rifampin	27 (69.2)	12 (30.8)	39 (32.5)
Trimethoprim-sulfamethoxazole	10 (62.5)	6 (37.5)	16 (13.3)
Erythromycin	47 (63.5)	27 (36.5)	74 (61.7)
Tetracycline	35 (62.5)	21 (37.5)	56 (46.7)
Gentamycin	55 (66.3)	28 (33.7)	83 (69.2)
Kanamycin	32 (68.1)	15 (31.9)	47 (39.2)
Clindamycin	42 (76.4)	13 (23.6)	55 (45.8)
Cefazoline	19 (79.2)	5 (20.8)	24 (20)
Ciprofloxacin	39 ()	12 (12)	51 (42.5)
Chloramphenicol	19 (79.2)	5 (20.8)	24 (20)

**Table 3:** Distribution of MDR patterns in MRSA and MSSA strains isolated from blood samples.

No. of antibiotics	Resistance pattern^a^	MRSA, *n* (%)	MSSA, *n* (%)	Total, *n* (%)
3	ERY, AMK, SXT	5 (17.2)	4 (13.8)	29 (24.2)
	ERY, AMP, AMK	10 (34.5)	10 (34.5)	

4	AMP, GEN, TET, AMK	8 (32)	9 (36)	25 (20.8)
	AMP, AMK, ERY, KAN	5 (20)	3 (12)	

5	A, CIP, ERY, SXT, AMP, AMK, ERY, GEN,	2 (18.2)	1(9.1)	11 (9.2)
	KAN, CEF, AMP, TET, GEN, RIF, AMK	3 (27.3)	5 (45.4)	

6	AMP, CIP, CEF, GEN, ERY, CLI	18 (75)	6 (25)	24 (20)

8	AMK, KAN, GEN, E, TET, CLI, RIF, AMP	5 (71.4)	2 (28.6)	7 (5.8)

10	AMP, KAN, GEN, E, TET, CLI, CHL, CIP, RIF, CFZ	12 (86)	2 (14)	14 (11.7)

11	ERY, AMP, KAN, GEN, CHL, CIP, RIF, CFZ, AMK, CLI, TET	4 (67)	2 (33)	6 (5)

12	AMP, AMK, E, GEN, KAN, CIP, CLI, ERY, TET, SXT, CFZ, RIF, CHL	3 (75)	1 (25)	4 (3.3)

aAMP: ampicillin; CLI: clindamycin; KAN: kanamycin; ERY: erythromycin; TET: tetracycline; CIP: ciprofloxacin; SXT: trimethoprim-sulfamethoxazole; GEN: gentamicin; CFZ: cephazolin; RIF: rifampicin; CHL: chloramphenicol. “a” refer to “a” before AMP in subtitle of the table.

**Table 4:** Distribution of different *coa* types in *S. aureus* strains obtained from 120 patients with bacteremia.

*Coa* types	MRSA 55 (46%)	MSSA 65 (54%)	Total
I	9 (100)	0 (0)	9 (7.5)
II	11 (34)	21 (66)	32 (26.7)
III	16 (45.7)	19 (54.3)	35 (29.2)
IVa	3 (100)	0 (0)	3 (2.5)
IVb	4 (100)	0 (0)	4 (3.3)
Va	2 (66.7)	1 (33.3)	3 (2.5)
VI	6 (50)	6 (50)	12 (10)
VII	3 (60)	2 (40)	5 (4.2)
VIII	8 (72.7)	3 (27.3)	11 (9.1)
X	3 (50)	3 (50)	6 (5)

## Data Availability

The data used to support the findings of the study are included in this article.
